# Benefits of Selected Physical Exercise Programs in Detention: A Randomized Controlled Study

**DOI:** 10.3390/ijerph10115683

**Published:** 2013-10-31

**Authors:** Claudia Battaglia, Alessandra di Cagno, Giovanni Fiorilli, Arrigo Giombini, Federica Fagnani, Paolo Borrione, Marco Marchetti, Fabio Pigozzi

**Affiliations:** 1 Department of Health Sciences, Italian University of Sport and Movement of Rome “Foro Italico”, Piazza Lauro De Bosis 15, Rome 00196, Italy; E-Mails: claudia.battaglia@uniroma4.it (C.B.); federica.fagnani@uniroma4.it (F.F.); paolo.borrione@uniroma4.it (P.B.); fabio.pigozzi@uniroma4.it (F.P.); 2 Department of Health Sciences, University of Molise, V. De Sanctis 1, Campobasso 86100, Italy; E-Mails: fiorilli@unimol.it (G.F.); arrigo.giombini@unimol.it (A.G.); marco.marchetti@unimol.it (M.M.)

**Keywords:** prison, aerobic and resistance training, cardiovascular disease, wellbeing

## Abstract

The aim of the study was to determine which kind of physical activity could be useful to inmate populations to improve their health status and fitness levels. A repeated measure design was used to evaluate the effects of two different training protocols on subjects in a state of detention, tested pre- and post-experimental protocol.Seventy-five male subjects were enrolled in the studyand randomly allocated to three groups: the cardiovascular plus resistance training protocol group (CRT) (*n* = 25; mean age 30.9 ± 8.9 years),the high-intensity strength training protocol group (HIST) (*n* = 25; mean age 33.9 ± 6.8 years), and a control group (C) (*n* = 25; mean age 32.9 ± 8.9 years) receiving no treatment. All subjects underwent a clinical assessmentandfitness tests. MANOVA revealed significant multivariate effects on group (*p* < 0.01) and group-training interaction (*p* < 0.05). CRT protocol resulted the most effective protocol to reach the best outcome in fitness tests. Both CRT and HIST protocols produced significant gains in the functional capacity (cardio-respiratory capacity and cardiovascular disease risk decrease) of incarcerated males. The significant gains obtained in functional capacity reflect the great potential of supervised exercise interventions for improving the health status of incarcerated people.

## 1. Introduction

Penal institutions are generally sick places [1]. Detention in overcrowded conditions [2] is generally coupled with unhealthy behaviors such as smoking, drug abuse, inactive lifestyle and irregular diet that lead to the development of a high rate of acute and chronic physiological and psychological diseases [3]. Prisoners respond to imprisonment, characterized by thepsychological pressures of incarceration, the social world of prison, beingdislocated from society, and the impact of the institution itself, with several physiological and mental problems. Prison inmate profiles reported poorer overall health status and a higher rate of healthcare utilization when compared to the general population [4]. In particular, imprisoned people usually have an increased risk of suffering from chronic debilitating conditions, co-infection with the HIV and hepatitis C virus and opioid dependence [5]. Moreover, health and psychological diseases increase medical personnel employment and pharmacological costs, increasing public expenditure [6]. In addition, incarceration has been associated with reduced physical activity. It has been widely demonstrated that sedentary people have a substantially increased risk of developing diabetes mellitus, heart diseases and other chronic disabilities [7]. Moreover, a history of incarceration is associated with a significantly elevated risk of future hypertension among inmates, thus the identification and the treatment of hypertension may be important in reducing risk of cardiovascular disease (CVD) in incarcerated individuals [8]. 

Considerable evidence accumulated in recent decades hasdemonstrated the protective effects of physical activity in both primary and secondary prevention of several chronic diseases. Several studies demonstrated that regular participation in endurance exercise training is an effective modalityto raise plasma high density lipoprotein (HDL) cholesterol levels, to reduce low density lipoprotein (LDL) cholesterol level [9] and to elevate triglyceride plasma concentrations [10] which are metabolic adaptations contributing to the reduced risk of coronary heart disease (CHD) [11]. Moreover, resistance training programscan lead to favorable health benefits in subjects at risk of developing type 2 diabetes, including reductions in central obesity and improved physical function [12,13], recently indicated asphysical activity guidelines for adults 150 min/week of moderate intensity or 75 min/week vigorous exercise (may also combine moderate and vigorous activity).The choice of the best training program, to improve inmates’ wellness should respond to the particular requirements of detention condition. The program may be conducted with light and easy available equipment, appropriate to the inmate antisocial behavior, which his related to difficulties in controlling violence and to protect them from potential safety risk Regular participation in physical activities increases, well-being and quality of life, and it has been reported that only supervised exercise training can improve these debilitating conditions as well as, the overall physical fitness of incarcerated people [5,14,15]. 

The aim of the present study was to determine if supervised cardiovascular plus resistance training could be preferable to the supervised high intensity strength, in order to improve prisoner health status. Considering that this special population have greatesthealth needs related to their sedentary lifestyle and their psychological profile, we hypothesized that cardiovascular plus resistance training could be the most effective training program on the cardio respiratory fitness, which promotes and maintains health, and an optimal caloric expenditure, although strength training is generally the prisoner preferred training. 

## 2. Methods

### 2.1. Study Design

This study was a repeated measures design intended to evaluate the effects of two different training protocols on fitness level and health status. The fitness level was evaluated on VO_2max_, strength and flexibility. The health status was evaluated on blood pressure, spirometric indices in subjects in a state of detention. At the beginning and at the end of the experimental protocol all subjects were tested to evaluate their fitness level and health status. 

### 2.2. Subjects

**Figure 1:**
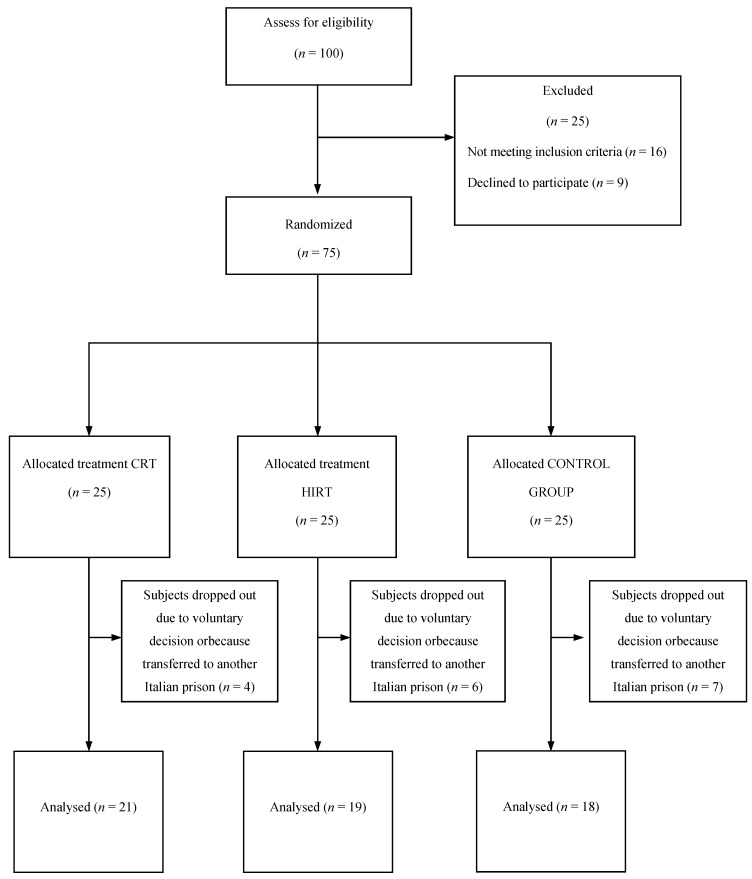
Flow chart.

The total sample size (*n* = 27) was estimated through an *apriori* power analysis. The analysis was carried out with the G*Power software (G*Power V 3.1.3 Franz Faul, Universität Kiel, Kiel, Germany), assuming anunivariate approach for between effects, within effects, and interactions. For the procedure were taken into account the following parameters (Cohen, 1988): effect size f = 0.33 (calculated from η_p_^2^ = 0.10—medium effect), α = 0.05, power = 0.80, and a correlation between repeated measures r = 0.50. This study took place in the Maximum Security Italian Prison of Larino (CB) and 75 subjects were enrolled in the study among male inmates of the prison. The inclusion criteria for participants included more than 1 year of detention and the age ≥50 years to allow the random assignment to the high intensity protocol. Subjects with severe orthopedic, cardiovascular or respiratory conditions that would preclude participation in an exercise program, or those with a medical condition listed in the American College of Sports Medicine [16] absolute exercise contraindications were also excluded. Subjects’ characteristics are shown in Table 1. Subjects were randomly allocated to one of the three treatment groups: the cardiovascular plus resistance training group (CRT) (*n* = 25; mean age 30.9 ± 8.9 years), the high-intensity strength training group (HIST) (*n* = 25; mean age 33.9 ± 6.8 years), and a control group (C) (*n* = 25; mean age 32.9 ± 8.9 years) receiving no treatment, in blocks of six (two subjects randomly assigned to each group), according to a sequence of computer-generated random numbers (SPSS version 16.0, Chicago, IL, USA). Subjects were informed on the rationale of the study, the use of data, and the issues and goals pursued. The study was designed in conformity with the Declaration of Helsinki and was approved by the local Ethics Committee (Protn. 27302_II/5 2011). All subjects signed an informed consent form before participating in the study. During the experimental period 17 subjects dropped out due to voluntary decision (*n* = 10) and to the fact that 7 subjects were moved to another prison. Thus, the final number of subjects evaluated was *n* = 58 (Figure 1).

**Table 1:** Sample characteristics (mean ± SD).

	CRT (*n* = 25)	HIRT (*n* = 25)	CONTROL GROUP (*n* =25)
Age (years)	30.1 ± 5.9	33.9 ± 6.8	32.8 ± 8.9
Prison term (years)	6.5 ± 4.8	9.7 ± 5.2	10.8 ± 7.2
Prisonterm completed (years)	3.7 ± 2.8	5.4 ± 4.7	4.3 ± 3.5
Prisonterm remaining (years)	2.8 ± 4.3	4.4 ± 2.2	6.5 ± 5.6

### 2.3. Experimental Procedures

**Figure 2:**
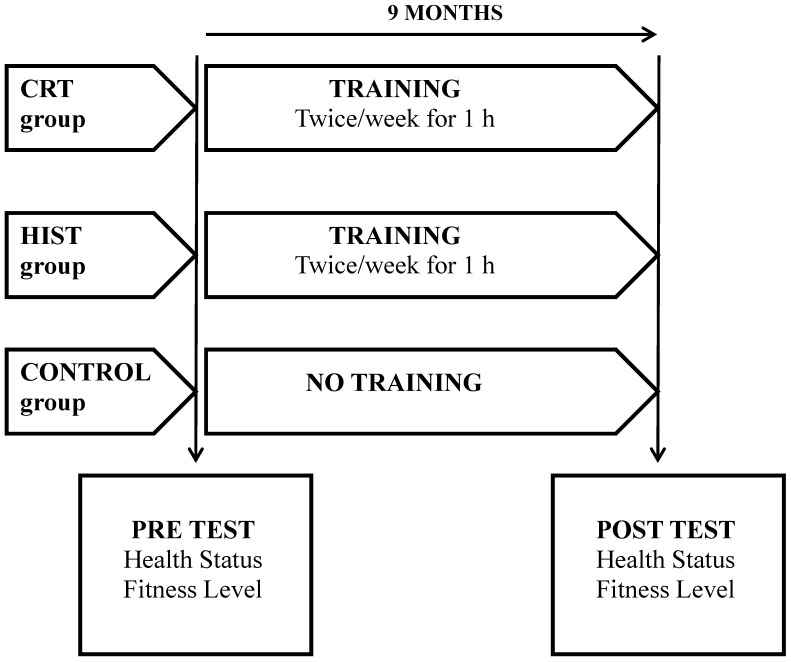
Experimental protocol.

The experimental groups (CRT and HIST) followed nine months of supervised fitness training protocols. For nine months, experimental groups took part one hour/twice weekly in the assigned training protocols. Every training session of the CRT protocol was composed as follows: 10 min of general warm up, 40 min of aerobic exercises alternated with resistance strength exercises and 10 min of stretching and muscle relaxing exercises. The aerobic training consisted of pedaling on a cycle ergometer or running on treadmill for 20 min. at 70% of the age-predicted maximum heart rate reserve (HRR). The duration and intensity of the sessions were gradually increased during the nine monthperiod, up to 45 min of activity at 80% of HRR by the end of the training program. Resistance training included exercises that engaged the major muscle groups (chest press, leg curl, leg press, leg calf rice, abdominal crunch, low back extension, arm curl, arm extension, and lateral pull down). All exercises were performed through the full range of motion normally associated with correct technique for each exercise. In the initial protocol subjects performed three sets with a resistance that allowed 12–15 repetitions (12–15 repetition maximum-RM) with 90 s rest [16]. Successively, the resistance used has been individually adjusted to allow the completion of 10–12 repetitions for three sets for the large muscle group exercises and two sets of the small muscle group exercises. The session training of the HIST protocol was composed as follows: 10 min of moderate bike warm up, 40 min ofanaerobic exercises alternated by maximal strength exercises and active recovery, and 10 min of cool down with relaxing exercises. The anaerobic training consisted of threesets of sprint training at 90% of the age-predicted maximum HRR, with 2 min. rest, and 30 s max effort sprint on bike alternatedwith3 min of easy pedaling. The duration and intensity of the sessions were gradually increased during the nine month-period, up tofivesets of sprint training at 95% of the age-predicted maximum HRR, with 2 min. rest, and 40 s max effort sprint on bike alternatedwith2 min of easy pedaling.Intensive strength training included exercises engaging the major muscle groups with a resistance that allowed 4–6 repetitions (6–8 repetition maximum RM) of triceps bench dips, hip lifts, prone planks (30 s hold), standing biceps curl, dumbbell (DB) squats, DB press, DB pullover, pushups standing DB lateral raise, DB split squat right and left leg, abdominal crunch. Concentric, Eccentric and Isometric muscle contractions were performed. Successively the resistance used has been individually adjusted to allow the completion of 1–6 repetitions (1–6 repetition maximum RM).During the experimental period, control subjects performed their habitual activities, receiving no physical activity treatment (Figure 2).

### 2.4. Health Status Measures

According to the medical team of the prison, all subjects were tested on three different days in the morning. During the testing session standard anthropometric measures, height and body weight, were assessed to calculate the measure of overall obesity, body mass index (BMI), calculated as weight in Kg/(height in meters) [3] according to standardized procedures [17]. 

All subjects underwent a clinical assessment. A resting blood pressure, systolic blood pressure (SBP) and diastolic blood pressure (DBP), and a pulse oximetry test (SpO_2_) were performed.The SpO_2_ was used to measure hemoglobin oxygen saturation. This is a non-invasive test that provides continuous, safe, and instantaneous measurement of blood oxygenation [18]. It is not only used to evaluate oxygen saturation of peripheral circulation but also to detect important metabolic disease [19]. Subjects performed a full spirometric test. The spirometric indices, cardiovascular fitness (CVF), forced expiratory volume in 1 s (FEV1) and Tiffenau Index were used.

CRT = Cardiovascular Resistant Training; HIST = High-Intensity Strength Training.

Blood sampling was obtained to assess total cholesterol (C-Total) and high-density lipoprotein (C-HDL), triglycerides plasma concentration, to estimate low-density lipoprotein plasma concentration (C-LDL), using the equation of [20] and to calculate the coronary heart disease (CHD) risk index, calculated from the ratio: C-total/C-HDL, used as a valid predictor of ischemic heart disease [21].

### 2.5. Fitness Level Testing Session

In accordance to the prison daily routine, the following tests were administered in the following order on the same time of the day, between 9 am and 12 pm.

The “Step test”, a test protocol for estimating maximal oxygen uptake which is the highest rate at which oxygen can be consumed, distributed and used by the body during exercise.The step test protocol consisted of stepping on a portable 10 inch (25.4 cm) high braced box (12 in (30.5 cm) wide, 18 in (45.7 cm) long and 10 in (25.4 cm) high) for three min per stage for a maximum of three stages. The stepping rates for the stages were 17, 26, and 34 steps per minute, respectively.During the last 30 s of the third minute of each stage of stepping heart rates were recorded at 2:30, 2:45 and 3:00 min. If the average of these three heart rates did not equal or exceed 65 percent of the age-predicted maximum heart rate (estimated as 220—age in years), then the subject was instructed to complete stage 2. Similarly, if the heart rates did not reach the 65 percent target level at the completion of stage 2, subjects then completed stage 3. Each stage was separated by at least one minute of sitting rest. Heart rate was monitored using a Polar RS300X Running Computer Heart Rate Monitorwith a WearLink Transmitter Polar (Polar Electro, Kempele, Finland), placed around the chest of the subject. The estimations of oxygen consumption were made using the equations, according to the last stage completed: stage 1 = 16.287 × body mass (kg)/1,000; stage 2 = 24.910 × body mass (kg)/1,000; stage 3 = 33.533 × body mass (kg)/1,000. The age-adjusted maximal oxygen consumption was then computed [22].

The “Sit and Reach test”, a common test that measures the flexibility of the lower back and hamstring muscles [23]. The test was repeated three times and the best value was considered. 

The “One min half sit-up test”, an abdominal muscular strength and endurance test [24] in which the total number of half sit-ups performed by each subject in one minute was recorded.

The “Push-up test”, an indicator of upper body and shoulder muscular strength and endurance, in which the total numbers of push-ups in one minute were recorded [25].

The “Arm Curl test” which measures upper body strength and endurance. The total number of complete movements undertaken using a 4 kg dumbbell of in 30 s was recorded [26].

The “Flamingo balance test”,performed to assess the ability to balance on a single leg after the “core” strength training. The total number of loss of balance in one minute was recorded [23].

The “10 × 5 shuttle run test”, to assess anaerobic power, speed and agility. The total time taken to complete 50 m was recorded [23].

### 2.6. Statistical Analyses

A one-way ANOVA was performed at the beginning of the experimental protocol to assess significant differences among groups at baseline. First a MANOVA test was run considering health status and fitness parameters, considering groups (CRT, HIST and control group) and training (pre and post) as independent variables and health status and fitness level parameters as dependent variables. Subsequently, a repeated measures 3 × 2 ANOVA was used to determine the variation of each health status and fitness level parameter among groups (CRT, HIST, control group), and within group (pre *vs*. post training), as factors. If significant main effects were found, a Student’s t-test was used as post hoc to identify the differences. The significance level was set *p* < 0.05. All statistics were performed using SPSS for Windows (version 16.0, SPSS inc., Chicago, IL, USA).

## 3. Results

Twenty three % of subjects dropped out the study in both the experimental protocols, becausethey did not adhere to or comply with the experimental protocol orwere moved to other Italian prisons (Figure 1). The one-way ANOVA revealed no significant differences among the three groups at baseline in terms of health and fitness status. MANOVA revealed significant multivariate effects on group (F_2.55_ = 2.889; *p* < 0.01) and group-training interaction (F_2.550_ = 1.994; *p* < 0.05). Considering each dependent variable of health status, the repeated measures 3 × 2 ANOVA showed a significant training effect for SpO_2_ (F_2.55_ = 3.973; *p* < 0.05), C-total (F_2.55_ = 8.082; *p* < 0.01), C-HDL (F_2.55_ = 19.774; *p* < 0.01), and CHD index risk (F_2.55_ = 8.612; *p* < 0.01). Significant training*group interactions were shown for BMI (F_2.55_ = 4.195; *p* < 0.05), SBP (F_2.55_ = 3.684; *p* < 0.01), DBP(F_2.55_ = 5.797; *p* < 0.01), C-HDL (F_2.55_ = 19.774; *p* < 0.01), triglycerides plasma concentration (F_2.55_ = 5.499; *p* < 0.01) (Table 2).

Considering fitness level, the repeated measure 3 × 2 ANOVA revealed significant group effect for sit and reach test (F_2.55_ = 5.349; *p* < 0.01), half sit-up test (F_2.55_ = 4.161; *p* < 0.05), push-up test (F_2.55_ = 3.913; *p* < 0.05), and arm curl test (F_2.55_ = 13.597; *p* < 0.01). Significant training effect was found for sit and reach test (F_2.55_ = 17.447; *p* < 0.01), half sit-up test (F_2.55_ = 14.012; *p* < 0.01), push-up test (F_2.55_ = 4.842; *p* < 0.05), arm curl test (F_2.55_ = 9.127; *p* < 0.01), and 10 × 5 shuttle test (F_2.55_ = 13.647; *p* < 0.01). Significant group*training interaction was found for step test (F_2.55_ = 8.469; *p* < 0.01), half sit-up test (F_2.55_ = 3.917; *p* < 0.05), push-up test (F_2.55_ = 5.529; *p* < 0.01), arm curl test (F_2.55_ = 8.254; *p* < 0.01), flamingo balance test (F_2.55_ = 12.688; *p* < 0.01) and shuttle test (F_2.55_ = 16.329; *p* < 0.01) (Table 3). 

## 4. Discussion

The priority of the prison system is to guarantee the social reintegration of inmates. Physical exercise plays animportant role as preventive tool in inmates’ re-education process. The main result of the present study was that nine-month supervised CRT could be considered the most effective training protocol to reach the best fitness level, with good effort tolerance. The present study promoted a cardiovascular plus resistance training program to verify the adherence of this particular population to prolonged exercise program, despite the low CRT effort tolerance. The feasibility of aerobic training protocol was confirmed by the inmate adherence to the exercise program. 

Whereas both the CRT and HIST protocols, produced significant gains in the functional capacity (cardio-respiratory capacity and CVD risk decrease) of incarcerated males, whereas an increase of triglycerides and BMI values over the same time period were observed in inactive inmates. Unhealthy weight gain, relieved in the control group, reflects an inactive lifestyle and wrong dietary behavior. Significant value improvement of SpO_2_ was also found after the training period in the experimental groups [27]. 

SpO_2_ is an effective screening tool for detecting significant lower extremity arterial diseases in patients with diabetes mellitus [28] and could predict mortality in systemic sclerosis-related interstitial lung disease (ILD) recognized as a distinct clinical entity in cigarette smokers [27,28]. In fact the 54% of the sample were smokers. The CRT protocol was effective for the significant decrease of BMI, SBP, DBP, CVD risk index and an increase of SpO_2_ near significance. The impact of regular aerobic exercise on C-LDL appears to be not significant, whereas endurance-trained subjects showed high C-HDL values compared to the sedentary population, as in the present study [29]. In addition, there are studies suggesting that resistance training, which is part of the CRT protocol in the present study, may also improve lipid and lipoprotein profiles [30]. Previous studies showed that intensive training programs significantly improved the total cholesterol levels, and endurance exercise consistently lowers triglycerides.

**Table 2:** Descriptive statistics (Mean ± SD) of health status variables.

Variable	CRT (*n* = 21)						HIST (*n* = 19)						CONTROL GROUP (*n* = 18)						η_p_^2^
	PRE			POST			PRE			POST			PRE			POST			
	Mean		SD	Mean		SD	Mean		SD	Mean		SD	Mean		SD	Mean		SD	
BMI (kg/m^2^)	29.6	±	4.1 **	28.0	±	3.5	27.8	±	3.8	27.5	±	2.6	28.3	±	2.7 **	28.7	±	2.7	0.07
SBP (mmHg)	124.7	±	8.1 **	113.0	±	11.9	121.0	±	8.9	119.3	±	11.0	119.2	±	6.4	120.8	±	16.6	0.08
DBP (mmHg)	73.3	±	7.0 **	67.3	±	7.0	74.0	±	5.1	70.0	±	4.1	68.5	±	9.0	71.9	±	7.5	0.06
SPO_2_ (%)	96.6	±	3.8	98.6	±	0.8 ^##^	97.8	±	1.5	98.6	±	1.0 ^##^	97.8	±	0.9	97.4	±	1.1	0.05
CVF (L)	4.8	±	0.7	4.9	±	0.7	4.9	±	0.7	5.0	±	0.6	4.8	±	0.4	4.9	±	0.5	0.13
FEV_1_ (L)	4.2	±	0.7	4.3	±	0.6	4.1	±	0.4	4.2	±	0.4	4.1	±	0.3	4.2	±	0.3	0.05
Tiffenauindex (%)	87.7	±	8.8	87.1	±	7.8	85.1	±	6.3	84.2	±	8.1	85.6	±	7.9	86.2	±	8.4	0.003
C-Total (mmol/L)	5.1	±	1.1	5.1	±	1.0	4.8	±	0.6 **	5.0	±	0.6	5.1	±	0.6	5.2	±	0.9	0.007
C-HDL (mmol/L)	1.2	±	0.3 **	1.4	±	0.2 ^#^	1.1	±	0.3 **	1.3	±	0.4	1.2	±	0.4	1.2	±	0.3	0.33
C-LDL (mmol/L)	2.9	±	1.1	2.8	±	1.0	2.8	±	0.8	2.8	±	0.8	3.0	±	0.6	3.1	±	0.9	0.002
CHD riskindex	4.6	±	1.8 **	3.8	±	1.1	5.0	±	2.6 *	4.3	±	1.8	4.7	±	1.9	4.4	±	1.8	0.17
Triglycerides (mmol/L)	2.1	±	0.5 *	2.0	±	0.4	2.1	±	0.3	2.0	±	0.3	1.9	±	0.4 *	2.0	±	0.4	0.06

* *p* < 0.05; ** *p* < 0.01 *vs*. post; **^#^** *p* < 0.05; **^##^** *p* < 0.01 *vs*. CONTROL GROUP (BMI: Body Mass Index; SBP: Systolic Blood Pressure; DBP: Diastolic Blood Pressure; SpO_2_: Pulse Oximetry test; CVF: Cardiovascular Fitness; FEV1: Forced Expiratory Volume in 1 s; C-Total: Total Cholesterol; C-HDL: High-Density Lipoprotein; C-LDL: Low-Density Lipoprotein; CHD: Coronary Heart Disease; CHD: Coronary Heart Disease).

**Table 3:** Descriptive statistics (Mean ± SD) of fitness level variables.

Variable	CRT (*n* = 21)						HIST (*n* = 19)						CONTROL GROUP (*n* = 18)						η_p_^2^
	PRE			POST			PRE			POST			PRE			POST			
	Mean		SD	Mean		SD	Mean		SD	Mean		SD	Mean		SD	Mean		SD	
Step test (mL/kg/min)	28.9	±	5.1 **	31.4	±	4.6 ^##^	28.8	±	4.2	28.5	±	4.1	27.0	±	4.2	26.5	±	4.3	0.07
Sit and Reach (cm)	23.8	±	6.3 **	29.0	±	6.9 ^##,†^	19.3	±	6.2	21.8	±	5.5	20.7	±	3.7	22.2	±	4.8	0.30
Half sit up test (n)	26.5	±	5.5 **	31.4	±	9.1^†^	29.4	±	6.8 **	42.8	±	12.8 ^#^	29.6	±	8.7	31.2	±	10.8	0.26
Push up test (n)	33.3	±	10.4 **	43.8	±	12.5 ^##^	25.7	±	10.2 **	35.7	±	13.9	30.7	±	13.3	24.8	±	16.8	0.10
Armcurl test (n)	22.9	±	3.9 **	28.9	±	4.8 ^##,†^	19.7	±	4.9 *	23.7	±	4.3 ^##^	20.1	±	3.7	17.8	±	5.5	0.19
Flamingo balance test (s)	3.1	±	1.8	2.1	±	2.3 ^##^	4.9	±	3.8 *	2.9	±	1.8 ^##^	3.2	±	2.9 **	5.8	±	3.3	0.003
10 × 5 shuttle test (s)	25.9	±	2.7 **	21.9	±	2.3 ^##^	27.3	±	3.0 **	23.8	±	1.3 ^##^	24.9	±	4.6 *	27.1	±	4.5	0.25

* *p* < 0.05; ** *p* < 0.01 *vs*. post; **^#^** *p* < 0.05; **^##^** *p* < 0.01 *vs*. CONTROL GROUP; **^†^** *p* < 0.01 *vs*. HIRT.

With regard to fitness level, the two experimental groups improved general strength, whereas the control group worsened balance and the agility level. Sedentary behaviors reduced strength, flexibility and coordination [31]. A significant increase in the aerobic capacity of the CRT group was detected. Despite that we did not directly measure VO_2max_, our findings is of clinical significance as we used an accepted test to estimate the aerobic fitness in adults [22]. The step test could reflect a decrease of HR response at higher workloads, objectively assessed, showing an improvement in aerobic condition. Moreover, an increase in HR response at high workloads represents a coronary artery disease prognosis [32]. Flexibility training, which help inmates to improve musculoskeletal health and to perform daily activities, was practice more in the CRT group than the HIST group, Our results are in agreement with previous studies, showing that cardiovascular plus resistance training, enhances defenses to chronic disease of a population living under the most difficult conditions of prison incarceration [5]. 

A significant improvement was observed in the upper body and abdominal dynamic strength endurance after both CRT and HIST protocols compared to the control group. The CRT and HIST groups were directly supervised by coaches, and it is recognized that supervised strength training causes major gains compared to unsupervised training [33]. Moreover, inmates need to feel as though their well-being has been taken into account; supervised exercise therefore may be more effective in improving functional capacity and well-being perception, with higher energy expenditure than self-managed training [34].

Despite the fact that no specific balance exercises were performed by the two experimental groups, intensive strength training of the HIST group improved not only general strength, but also balance due to a better increase of core muscle strength and consequently of postural stability [35]. Muscle strength, coordination and dynamic balance trained in both the two experimental protocols improved the agility, assessed by a shuttle test, as it is demonstrated in the literature [31]. We observed a significant increase of anaerobic power assessed by shuttle test, in both the two experimental groups compared to the control group. If intense physical exercise is carried out correctly under the supervision of specialized trainer, it can contribute to the enhancement in the quality of life, as well as to the health of groups, such as previous studies demonstrated [36]. 

In conclusion, CRT allows to reach better aerobic capacity, which promotes and maintains health, and an optimal caloric expenditure [37]. This training protocol guarantees a strength level sufficient to sustain daily living that is made up of mostly sub-maximal-strength tasks and a good level of flexibility and agility that could help this special population to reduce injuries and consequent chronic diseases [38]. Moreover, CRT requireseasy available and light equipment that protect from potential safety risk to other inmates, if some equipmentcould be used as a weapon in a fight. HIST protocol equipment are more onerous in terms of costs and management requirements, based onprison availability.

To date, little research efforts have focused exercise training interventions on improving the health status and the well-being, of prison inmates, a population that deserves the consideration of the scientific community. Previous study highlighted that the efficacy of physical exercise could be a complementary therapy in several medical treatments [15]. On the other hand physical activity, teaches discipline, record-keeping and goal-setting, employs the inmate leisure time reducing boredom and burning off tension [39,40].

In light of the relationship between regular and supervised physical activity and inmates’ health, prison administration staff should promote this healthy inmate behavior. Healthcare providersshould encourage inmates to participate in aerobic recreational activities and to increase physical activity during their daily routine thatresults in significant weight loss and improved cardiovascular capacity. In particular, aerobic exercises combined with recommendations to consume a were balanced diet could have beneficial effects on lipid and lipoprotein concentrations in adults [41]. 

Our study is not without methodological limitation: the results of the study are referable only to prisoners with the age ≥ 50 years. This age range allows the inmates to participate to HIST protocol. No direct assessment of several aerobic capacity (VO_2max_), was carried out. Accordingly, we performed all testing procedures inside the penitentiary centre with relatively low adherence to training program 57% a less of this unmotivated population, living under very difficult conditions compared to free individuals. However, the significant gains obtained in functional capacity, reflect the great potential of exercise interventions for improving the health status of incarcerated people.

## 5. Conclusions

Current results suggest that supervised physical activity improved fitness and health status in prisoners. Prisoner’s health care is completely relianton the nation state. It was expected that a good health status could reduce medicine and medical assistance usein prison, with a substantial decrease of public spending. Moreover, physical activity could be a useful leisure time occupation, which is a serious problem in prison, and could properly prepare for social reintegration. 

Prisoners showed an increase of triglycerides and BMI values due to inactive lifestyle and wrong dietary behavior. Aerobic plus resistance training was effective for the significant decrease of BMI, systolic blood pressure, diastolic blood pressure, coronary heart disease (CHD risk index), a valid predictor of ischemic heart disease, and an increase in pulse-oximetry test (SpO_2_). Whereas intensive training program significantly improved the total cholesterol levels. Effects of the different training protocols have been monitored to find the most useful and simple method to introduce it in other prison. 

Thus, it might be interesting exploring the correlation between the physical activity adherence and the decrease of crimes committed by inmates after release.
